# Metal–Organic Framework (MOF)-Embedded Magnetic Polysaccharide Hydrogel Beads as Efficient Adsorbents for Malachite Green Removal

**DOI:** 10.3390/molecules30071560

**Published:** 2025-03-31

**Authors:** Lei Cheng, Yunzhu Lu, Peiyi Li, Baoguo Sun, Lidong Wu

**Affiliations:** 1 Beijing Engineering and Technology Research Center of Food Additives, Beijing Advanced Innovation Center for Food Nutrition and Human Health, Beijing Technology and Business University, Beijing 100048, China; chenglei@btbu.edu.cn (L.C.); lyz754784549@163.com (Y.L.); sunbg@btbu.edu.cn (B.S.); 2 Key Laboratory of Control of Quality and Safety for Aquatic Products, Ministry of Agriculture, Chinese Academy of Fishery Sciences, Beijing 100141, China; lippy1998@163.com

**Keywords:** alginate, MMOF hydrogel, selective separation, malachite green, removal

## Abstract

Sodium alginate is a polysaccharide compound extracted from natural plants that has been successfully prepared as a hydrogel for adsorbing and removing pollutants. However, the selectivity of alginate-based hydrogels to malachite green (MG) dyes and the stability of alginate-based hydrogels in air cannot meet requirements. Herein, metal–organic frameworks (MOFs) are embedded into a magnetic hydrogel to create magnetic MOF hydrogel (MMOF hydrogel) microspheres with high adsorption capacity. The morphology and physical properties of the MMOF hydrogel microspheres were characterized by scanning electron microscopy and optical microscopy. Under optimized adsorption conditions, the adsorption rate of MG reached 96.5%. The maximum adsorption capacity of the MMOF hydrogel for MG was determined to be 315 mg·g^−1^. This highly efficient magnetic adsorbent for dye removal has considerable potential for rapidly removing toxic contaminants from aquatic food matrices for high-throughput sampling pretreatment, which has the potential for rapid, green, large-scale environmental remediation in the future.

## 1. Introduction

Alginate is an unbranched, linear anionic, water-soluble, and natural extraction polysaccharide composed of repeating (1,4)-linked α-l-guluronic and β-d-mannuronic acids [1]. Benefiting from its hydrophilicity, nontoxicity, and cost-effective properties, alginate hydrogel has great application as an adsorbent. The primary focus in the development of hydrogels has been on creating super adsorbents, such as semi-interpenetrating networks and cross-linked binary graft copolymers [2,3,4]. The hydrogel adsorbents have been successfully designed to remove micro-pollutant [5,6,7,8]. However, most hydrogels are ineffective in removing organic dyes and have limited selectivity for organic pollutants.

Previous researchers introduced a polyacrylamide hydrogel incorporating radially symmetric cyclodextrin, wherein the transport metrics—such as selectivity, rate, and concentration limits—of the target organic molecule are intricately linked to the robustness of the host–guest interaction [9,10]. While this hydrogel exhibits remarkable selectivity for organic molecules, its fabrication relies on spatially localized hydrolysis and amide coupling reactions to establish the chemical gradient [11,12]. This process is cumbersome and impractical for real-world applications [13]. Consequently, there is a pressing need to develop a hydrogel that is simple to prepare and can spontaneously and selectively remove contaminants, thereby enhancing its practical utility.

In this work, the recyclability and portability of hydrogel are enhanced using magnetic materials, while its adsorption performance is improved with MOF materials. A novel adsorbent material for removing MG is introduced. Magnetic hydrogels offer rapid and efficient separation due to their magnetic responsiveness, enabling portability, easy recovery, and re-usability. The spherical hydrogel encapsulates magnetic particles within a defined area, endowing the adsorbent with strong magnetic properties. Furthermore, MOFs are embedded in a magnetic hydrogel as a flexible scaffold to enhance its adsorption performance for MG dye. We also investigated the impact of the MOF weight ratio and adsorbent dosage on the MG dye adsorption rate. Because of its high separation efficiency and flexible properties, MMOF hydrogel is applied to in-site MG dye removal on the aquatic tissue surface.

## 2. Results and Discussion

### 2.1. Characterization of MMOF Hydrogel

**Figure 1:**
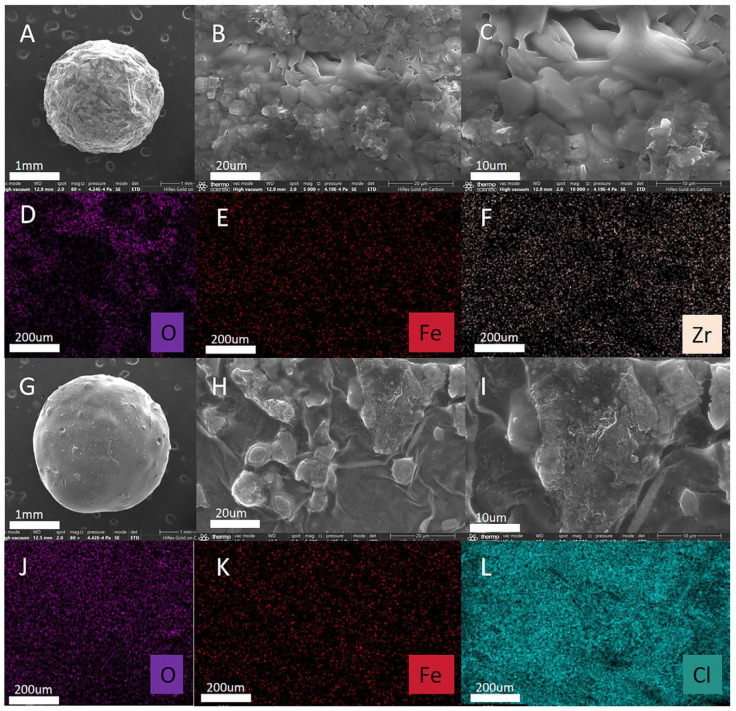
Characterization of MMOF hydrogel SEM images. (**A**–**C**) Scanning electron microscopy (SEM) characterization of MMOF hydrogels. (**D**–**F**) MMOF hydrogel energy dispersive X-ray spectroscopy (EDS) element mapping. (**G**–**H**) Scanning electron microscopy (SEM) characterization of MMOF-hydrogel-bead-adsorbed MG dye. (**J**–**L**) Energy dispersive X-ray spectroscopy (EDS) elemental mapping after adsorption of MG dyes by MMOF hydrogels.

The morphologies of the MMOF hydrogels were analyzed using SEM. Figure 1 illustrates the structural changes before and after the adsorption of MG. Figure 1A,G show that the MMOF hydrogel composite microspheres exhibited a well-defined structure with distinct surface grooves before adsorption. This morphology contributes to forming a micro-porous structure, which enhances the specific surface area and provides numerous adsorption sites for MG. Following adsorption, a marked increase in MG uptake was observed, accompanied by a reduction in the number and depth of the surface gullies. As shown in Figure 1B,C,H,I, notable alterations in the surface morphology were evident, with the surface becoming significantly smoother after MG adsorption, indicating successful adsorption onto the microsphere surface. Elemental mapping, presented in Figure 1D–F, confirmed the distribution of O, Fe, and Zr on the microsphere surface before adsorption. Additionally, after MG adsorption, the presence of O, Fe, and Cl on the surface of the microspheres, as shown in Figure 1J–L, further corroborated the successful adsorption of MG. After MG adsorption, the composition of elements on the surface of the microspheres changed because of the ion exchange reaction between H^+^ in the MMOF hydrogel and Cl^−^ in the MG dye so as to achieve adsorption.

### 2.2. Optimization of Adsorption Conditions

#### 2.2.1. Optimization of Adsorption Materials

**Figure 2:**
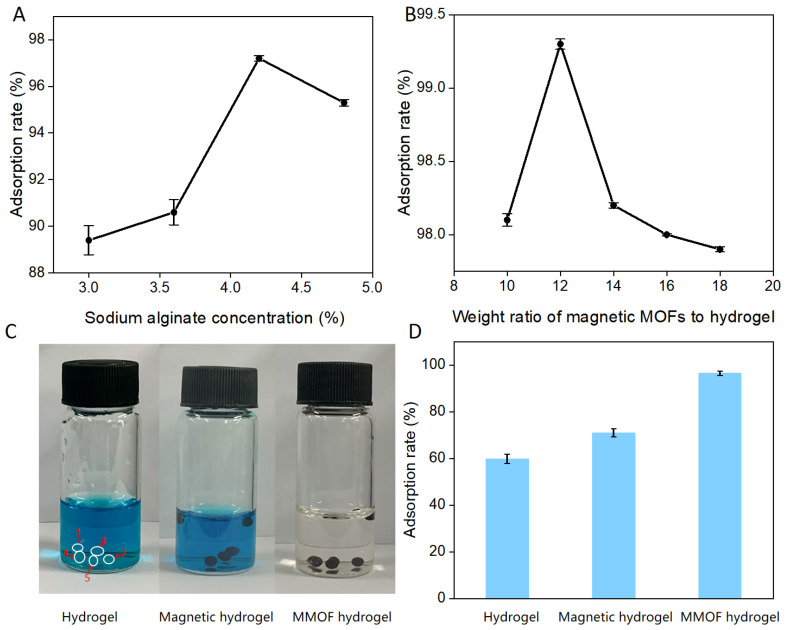
Optimization of MMOF hydrogel microspheres. (**A**) Influence of sodium alginate concentration. (**B**) Weight ratio of magnetic MOFs to hydrogel. (**C**) Photos of pure hydrogels, magnetic hydrogels, and MMOF hydrogels for dye adsorption of MG. (**D**) Adsorption rates of hydrogels, magnetic hydrogels, and MMOF hydrogels.

SA is an important raw material for preparing MMOF hydrogel, as it is vital to improving the stability and portability of microspheres. The adsorption of MG by microspheres was significantly enhanced by optimizing the concentration of SA. As shown in Figure 2A, the adsorption was recorded by varying the percentage concentration of SA between 3% and 4.8%. When the concentration of SA was 4.2%, the decolorization rate of MG was the highest.

The adsorption capacity was found to be associated with the weight ratio of magnetic MOFs to hydrogel. Figure 2B shows that the decolorization rate decreases as the mass of the magnetic MOFs increases within a range of 10 and 18. The decolorization rate reached 99.3% when the weight ratio of magnetic MOFS to hydrogel was 12, indicating that the MMOF hydrogel displayed the best adsorption of MG under this condition.

As shown in Figure 2C,D, the adsorption of MG was compared between MMOF hydrogel, pure hydrogel, and magnetic hydrogel under the same conditions. In Figure 2C, it is difficult to clearly see the light color of the non-magnetic MOF hydrogel in the figure, so it is marked with a white circle. The MMOF hydrogel with MOFs achieved a decolorization efficiency of 96.5%. On the other hand, the decolorization efficiency of the MMOF hydrogel without magnetic MOFs and with Fe_3_O_4_ only achieved decolorization efficiencies of 59.9% and 71%, respectively.

The adsorption effect of MMOF hydrogel for MG was significantly higher than that of hydrogel microspheres and magnetic hydrogel microspheres. The results revealed that combining magnetic MOFs and hydrogel was highly effective and addressed the low adsorption of MG with traditional hydrogels.

#### 2.2.2. Optimization of Adsorption Environment

**Figure 3:**
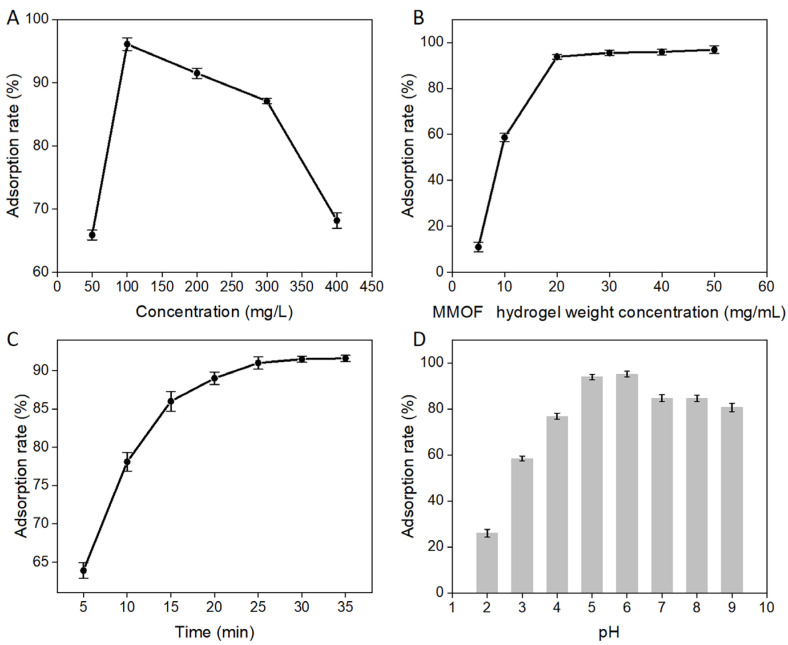
Optimization of adsorption conditions for MG dyes using MMOF hydrogel microspheres. (**A**) The effect of different initial concentrations of MG on the adsorption properties of MMOF hydrogel. (**B**) When the concentration of MG solution is 100 mg/mL, and the volume is 10 mL, the MMOF hydrogel with different weights influences the decolorization rate. (**C**) When the concentration of the MG solution is 100 mg/mL, time affects the adsorption properties of the MMOF hydrogel. (**D**) When the concentration of the MG solution is 100 mg/mL, the pH values of different MG solutions affect the adsorption properties of MMOF hydrogel.

The adsorption of MG by the MMOF hydrogel was examined using the following concentrations (Figure 3A): 50 mg/L, 100 mg/L, 200 mg/L, 300 mg/L, and 400 mg/L. Furthermore, the pH value of the MG solution was adjusted to 6, while 200 mg of MMOF hydrogels was added. The adsorption rate was examined after oscillating the solution for 30 min. The adsorption effect of the material was the largest when the MG concentration was 100 mg/L. However, as the concentration of MG increased, additional MG molecules competed for the adsorption sites on the surfaces of the MMOF hydrogels, leading to a reduced decolorization rate.

As shown in Figure 3B, the adsorption rate was examined for various MMOF hydrogel adsorbent weights, keeping the MG solution concentration at 100 mg/mL, the pH at 6, and the volume at 10 mL. The following adsorbent weights were tested: 50 mg, 100 mg, 200 mg, 300 mg, 400 mg, and 500 mg. When the MMOF hydrogel weight concentration was over 20 mg/mL, the decolorization rate reached a constant value of about 95%. This was because the adsorption sites of the MMOF hydrogel (20 mg/mL) for MG almost reached a state of equilibrium. Even though the weight of the MMOF hydrogel increased, the adsorption capacity for MG reached a plateau, indicating that the available adsorption sites were saturated or that equilibrium had been achieved. By changing the conditions, the interaction between the MMOF hydrogel and the solvent to be adsorbed was increased by flipping, thus reducing the adsorption time to 30 min (Figure 3C), with the MMOF hydrogel reaching the saturated state and the decolorization rate reaching 91.5%.

The pH value of the MG solution significantly influenced the adsorption efficiency of the MMOF hydrogel. This affected the ionic state of the MG solution and the positively and negatively charged functional groups in the MMOF hydrogel [14,15,16]. The effect of pH on the adsorption efficiency of the MG solution was examined by varying the pH between 2 and 9 in the presence of 200 mg of MMOF hydrogel.

As shown in Figure 3D, there is a large concentration of H^+^ in the solution in a pH range of 2–4. These H^+^ ions compete with MG by combining with the negative charge of the functional group in the MMOF hydrogel [17,18,19]. However, at pH 7–9, the carboxyl group on the MMOF hydrogel is ionized, thereby decreasing the adsorption rate of MG. Consequently, there was a significant decrease in the adsorption rate of MG. The optimal adsorption condition was recorded at pH 6.

### 2.3. Dynamic Adsorption Model

**Figure 4:**
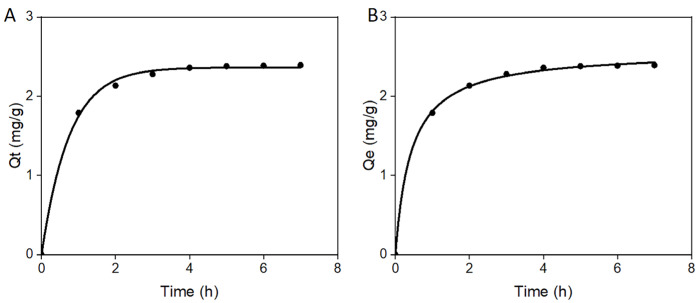
Dynamic adsorption model. (**A**) Pseudo-first-order model. (**B**) Pseudo-second-order model.

The kinetics of the adsorption of MG by the MMOF hydrogel was studied by adopting a quasi-first-order kinetic model [20,21,22] and a quasi-second-order kinetic mode [23,24,25,26] (Figure 4). The relevant parameters are listed in Table 1 and Table 2. The relationships shown in Equations (1)–(3) represent the quasi-first-order kinetic model, the quasi-second-order kinetic model, and the parameters of the intra-particle diffusion model.

(1) $$\log\left(\mathrm{Qe}-\mathrm{Qt}\right)=\log\left(\mathrm{Qe}\right)-\left(K_{1}/2.303\right)\times t$$

(2) $$t/\mathrm{Qt}=1/\left(K_{2}\times {\mathrm{Qe}}^{2}\right)+t/\mathrm{Qe}$$

(3) $$\mathrm{Qt}=K_{3}\times t^{1/2}+C$$

In this formula, Qe represents the adsorption amount at equilibrium time in mg/g; Qt represents the adsorption capacity at time t in mg/g; C is the boundary layer thickness; K_1_ and K_2_ represent the quasi-first-order and the quasi-second-order kinetic rate constants, respectively; K_3_ represents the parameters of the intra-particle diffusion model; and t represents time [27,28,29,30].

Table 1 shows that the correlation coefficient, R^2^, values of the quasi-first-order kinetic model and the quasi-second-order kinetic model were calculated to be 0.007 and 0.999, respectively. This suggested that the quasi-second-order kinetic model exhibited a better fit than the quasi-first-order kinetic model. Moreover, the rate constants of both the quasi-first-order kinetic model and the quasi-second-order kinetic model were over 0.9, suggesting that both models could describe the adsorption kinetics of MG via MMOF hydrogels. Since the rate constant of the quasi-first-order kinetic model was higher than the quasi-second-order kinetic model, and the Qe value was closer to the true value, it was concluded that the adsorption process of MMOF hydrogel for MG was mainly dominated by physical adsorption.

Table 2 shows the in-particle diffusion kinetic model used to study the control steps of the adsorption rate at each stage. If C was not 0, the fitted curve did not pass through 0, thereby suggesting that internal diffusion was not the only rate-limiting step and included both in-particle diffusion and surface diffusion. Furthermore, K_1_ > K_2_ > K_3_ indicated that the adsorption site on the surface of the MMOF hydrogel was occupied by adsorbed substances, slowing down the adsorption rate and finally reaching equilibrium.

### 2.4. Application of MMOF Hydrogels to Pigment Removal

**Figure 5:**
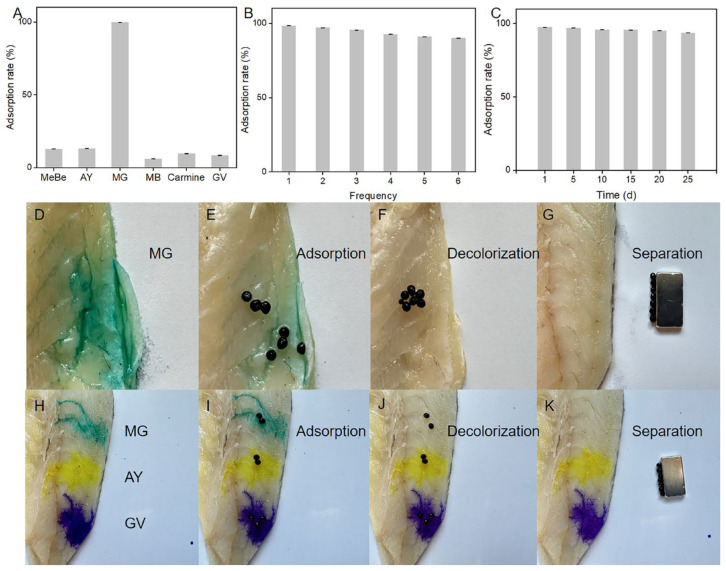
Application of MMOF hydrogels to pigment removal. (**A**) Comparison of the adsorption capacity of MMOF hydrogel toward different dyes (MG, acridine yellow, methylene blue, carmine, and crystal violet dyes). (**B**) Reusability assessment of MMOF hydrogel: Adsorption rate toward MG dye over 6 consecutive adsorption–desorption cycles is over 90%. (**C**) Durability evaluation of MMOF hydrogel: Adsorption rate toward MG dye sustained at over 90% after 25 days. (**D**) Images of MG dye on the surface of fish tissue. (**E**) MMOF hydrogel beads adsorb MG dye to the surface of the fish. (**F**) After adsorption, the surface of the fish tissue becomes colorless. (**G**) There is no residual adsorbent on the surface of the fish tissue after magnetic separation. (**H**) Images of MG, AY, and GV dyes on the surface of fish tissue. (**I**) MMOF hydrogel beads placed on MG, AY, and GV dyes on the surface of the fish. (**J**) After adsorption, the original MG dye on the surface of the fish tissue becomes colorless, but AY and GV dyes still exist on the fish surface. (**K**) After magnetic separation, there is no residual GM, AY, or GV on the surface of the fish tissue. Scale: 1 cm.

Five kinds of pigments, methyl blue, acridinium yellow, methylene blue, carmine red, and gentian violet, were used to study the adsorption effect of MMOF hydrogel (Figure 5A). The adsorption capacity of MMOF hydrogel for MG was found to be much greater compared with that of the other five pigments. This result suggested that MMOF hydrogel offered selectivity for MG. Reproducibility and stability are critical factors required for novel composite materials. Six extraction–analysis cycles were tested (Figure 5B) to explore the reproducibility of the prepared MMOF hydrogel. The adsorption rates for all cycles were found to be over 90%. Thus, MMOF hydrogel exhibited good reproducibility, reduced experimental costs, and met the requirements of environmental protection.

Next, the experiment aimed to explore the stability of the composite material. MMOF hydrogels were dried and sealed at 60 °C for 25 days, and MMOF hydrogel microspheres (200 mg) were removed every 5 days and placed in 10 mL of solvent for adsorption and elution. With an increase in storage time, the adsorption efficiency and elution rate of the material were found to be stable (Figure 5C), with a decolorization rate of over 90%, suggesting that the material possessed strong stability. In an article on an MG detection method studied by other researchers, adsorption materials such as MOF needed to be stored in methanol and other solutions, and methanol needed to be completely removed before detection. This process is too cumbersome, and the stability is poor. MMOF hydrogel makes up for this defect. As proof of this fact, we show the in situ MG removal from fish tissue surface in Figure 5D–G. After magnetic separation, the tissue surface of the fish is free of MG dye and residual adsorbent. The application of MMOF hydrogels improves dye removal efficiency. MG, AY, and GV dyes are dropped on the surface of the fish, and then, MMOF hydrogels are placed on the MG, AY, and GV dyes. After 30 min of adsorption, the MG dyes on the surface of the fish have been completely removed after magnetic separation, while the AY and GY dyes are still on the surface of the fish. Therefore, MMOF hydrogels are highly selective to MG. In terms of selectivity to malachite green, MMOF hydrogel has stronger selectivity to MG than other materials and will not be interfered with by other factors.

## 3. Materials and Methods

### 3.1. Materials

The following chemical reagents were used in the experimental procedures: iron (III) chloride hexahydrate (FeCl_3_·6H_2_O), iron (II) chloride tetra-hydrate (FeCl_2_·4H_2_O), *N*,*N*-dimethylacetamide (DMF, 99.5%, AR), ammonium hydroxide, ethyl alcohol (99.5%, AR), sodium alginate (SA) with a molecular weight of 216.1215 g/mol, 1% viscosity 550 mPa.s, a β-D-mannuronic acid-to-α-L-glucuronic acid ratio of 1:1, benzoic acid (BA), PVP (Mw = 40,000), tetrakis(4-carboxyphenyl) porphyrin (TCPP, 97%), acrylamide (AM), ammonium per-sulfate (APS), *N*,*N*-methylenebisacrylamide (BIS), calcium chloride (CaCl_2_), N,N,N_9_,N_9_-tetramethylethylenediamine (TEMED, AR), sodium citrate, zirconyl chloride, acetonitrile (AR), methanol (AR), ammonia (AR), and MG. these reagents were purchased from Macklin (Beijing, China). Fish meat was purchased at the supermarket.

### 3.2. Instruments

The instruments used in this study were as follows: a vortex oscillator VM-500 pro (JOANLAB, Huzhou, China), a magnetic stirrer and oil bath pan (Li Chen, Shanghai, China), a UV–visible spectrophotometer (Shimadzu UV-3600Plu, Kyoto, Japan), a centrifuge (Xiangyi, China), a pH meter, a scanning electron microscope (Thermo Fisher Quattro S, SEM, Eindhoven, The Netherlands), and a nanoparticle size and zeta potential analyzer (Zetasizer Nano ZS90, Malvern, UK).

### 3.3. Synthesis

#### 3.3.1. Synthesis of Fe_3_O_4_@MOF

Under nitrogen conditions, FeCl_3_·6H_2_O and FeCl_2_·4H_2_O were dissolved in a mixed solution of water and ethanol, followed by the addition of ammonia water. After centrifugation, Fe_3_O_4_ nanoparticles with an average particle size of 95 nm were collected. We added PVP powder to the Fe_3_O_4_ and shook and then added TCPP, ZrOCl_2_·8H_2_O, BA, and Fe_3_O_4_@PVP. We then took the oil bath and washed it with DMF to obtain Fe_3_O_4_@PVP. The average particle size of the MOF was 1100 nm. Fe_3_O_4_@PVP@MOF was stored in a chromatographic-grade methanol solution. The nanoparticle size and zeta potential analyzer was used for particle size detection for both Fe_3_O_4_ and Fe_3_O_4_@PVP@MOF.

#### 3.3.2. Synthesis of the MMOF Hydrogel

We added Fe_3_O_4_@MOF-545 (5.3 mg/mL), 19% AM (0.49 mL/mL), 1.5% APS (12 μL/mL), and 2% BIS (53.8 μL/mL) to syringe A. Then, 4.2% SA solution (0.45 mL/mL) was added to syringe B for coupling with a needle connector, and then, 20 μL of TEMED was added. The mixed solution was slowly dropped into a CaCl_2_ (5 mol/L) solution by hand with a syringe, and the dropping rate of the solution was controlled to drop 1 drop every 1 s. If it was too fast, the microsphere adhered, and if it was too slow, the solution in the needle formed a colloid and could not be dropped into the CaCl_2_ solution, and it was stirred magnetically for 1 h to obtain MMOF hydrogel. The volume ratio added was the ratio of the solution before adding TEMED.

#### 3.3.3. Synthesis of the Pure Hydrogel

We added AM, APS, and BIS to syringe A and SA to syringe B—their concentration and dosage were the same as those of the MMOF hydrogel—followed by the addition of 20 μL of TEMED. The hydrogel microspheres were prepared by injecting the syringe into an anhydrous solution of CaCl_2_ (5 mol/L), which was magnetically stirred for 1 h, followed by filtration and drying.

#### 3.3.4. Synthesis of the Magnetic Hydrogel

Except for adding Fe_3_O_4_ powder (30 mg), the preparation conditions for the rest of the hydrogels are the same as those of pure hydrogel.

### 3.4. Adsorption of MG by MMOF Hydrogel

To obtain a standard curve to adsorb MG dye and evaluate the accuracy of the measurement method, first, MG (10 mg) was dissolved in 100 mL of ultra-pure water to obtain a 100 mg/L MG solution. This stock solution was diluted with ultra-pure water to obtain MG solutions with concentrations of 1, 2, 3, 4, and 5 mg/L. Next, the adsorbance of these solutions was measured using the UV–visible spectrophotometer (616 nm), obtaining adsorbance values corresponding to concentrations of 0.032, 0.066, 0.097, 0.134, and 0.171, respectively. After conducting 5 repeated experiments, we obtained the adsorption standard curve with the following linear equation: a = 0.035 C − 0.004, R^2^ = 0.999 (A: adsorbance, C: concentration of MG solution, and R^2^: correlation coefficient; 0.035 and 0.004 are correlation coefficients calculated based on adsorbance and solution concentration). The relative standard deviation was 1.47%.

### 3.5. MG Removal Using MMOF Hydrogels

**Figure 6:**
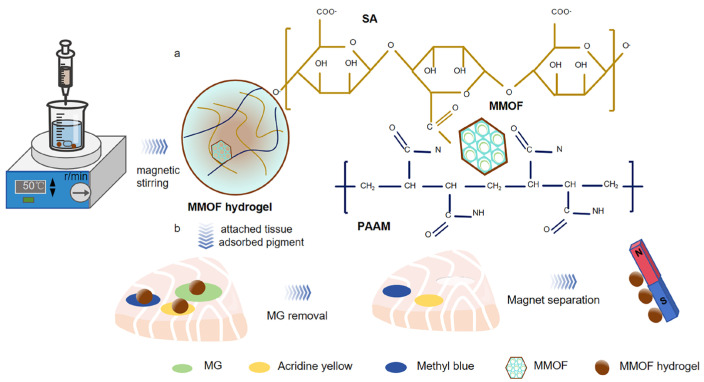
MMOF hydrogel microspheres adsorb MG dye on the surface of fish tissue. (**a**) MMOF hydrogel concept map. (**b**) MMOF hydrogels attached to fish surfaces for different dye removal effects and magnet separation procedures.

MMOF hydrogels can be attached to tissue surfaces, and MG can be accurately, rapidly, and selectively removed (Figure 6). The maximum adsorption rate of MG can be achieved by using different solvent concentrations, adsorption times, pH values, material qualities, etc. A 100 mg/L MG solution (10 mL) was adsorbed on 200 mg of MMOF hydrogel and detected using the UV–visible spectrophotometer. The rate of decolorization was calculated using the following Formula (4).

(4) $$X=\left(C_{0}-C_{1}\right)/C_{0}\times 100\%$$

C_0_ is the adsorbance of the original solution, C_1_ is the adsorbance of the adsorbed solution, and X is the rate of decolorization.

The MG in fish and shrimp was removed by MMOF hydrogel microspheres.

### 3.6. Supplementary Experimental Conditions

Exploring the optimization of MMOF hydrogel’s conditions was carried out under the condition that the MG dye concentration was 100 mg/mL and the pH was 6. Optimizing the conditions and adsorption kinetics model of MG dye adsorption on MMOF hydrogel was carried out under the condition of 4.2% SA, and the weight ratio of magnetic MOFs to sodium alginate was 12. When studying the selective adsorption of MG dyes on MMOF hydrogel, the concentration of the AY, MeBe, MB, carmine, and GV dyes was 100 mg/L, and the pH was 6. The conditions of the MMOF hydrogel material were the same as those of the adsorption kinetics model.

### 3.7. Data Processing Methods and Software

The data processing methods used in this article include data organization and data visualization. Data organization is the process of classifying, sorting, and encoding raw data to make them easier to analyze. Data visualization is the visual display of the distribution and characteristics of data through charts, and this article mainly uses bar charts and line charts. The software used for statistical processing was Origin 2018 (Reachsoft, Beijing, China).

## 4. Conclusions

In this study, we successfully synthesized MMOF hydrogels. These hydrogels encapsulate magnetic particles in a targeted manner, significantly enhancing the adsorbent’s magnetic properties. The MOFs are embedded within the magnetic hydrogel, acting as a flexible scaffold that reinforces the hydrogel’s structure and boosts its adsorption efficiency for MG. The MMOF hydrogels are highly selective for MG and can be easily separated from complex environments. They are specifically designed to remove MG from the surface of aquatic tissues in situ. This research has developed a magnetic adsorption material that exhibits strong selectivity, separation capabilities, and adsorption performance for MG. The controlled separation of MMOF hydrogel has the potential for automated applications, making it an effective ecological and high-throughput pretreatment solution for aquatic products.

## Figures and Tables

**Table 1:** Kinetic parameters.

Pseudo-First-Order Model			Pseudo-Second-Order Model		
K_1_	Qe (mg/g)	R^2^	K_2_	Qe (mg/g)	R^2^
1.342	2.364	0.997	0.923	2.573	0.999

**Table 2:** Parameters of the intra-particle diffusion model.

The First Stage			The Second Stage			The Third Stage		
K_1_	C_1_	R_1_^2^	K_2_	C_2_	R_2_^2^	K_3_	C_3_	R_3_^2^
0.676	1.134	0.951	0.201	1.94	0.817	0.032	2.308	0.999

## Data Availability

The original contributions presented in the study are included in the article, further inquiries can be directed to the corresponding author.
